# Metal Bionanohybrids against Microbiologically Influenced Corrosion (MIC) Consortia

**DOI:** 10.3390/nano14171376

**Published:** 2024-08-23

**Authors:** Clara Ortega-Nieto, Maria Salta, Nanni Noël-Hermes, Jose M. Palomo

**Affiliations:** 1 Instituto de Catálisis y Petroleoquímica (ICP), CSIC, c/Marie Curie 2, 28049 Madrid, Spain; clara.ortega@csic.es; 2 Endures B.V., 1781 AT Den Helder, The Netherlands; 3 School of Biological Sciences, Faculty of Science and Health, University of Portsmouth, Portsmouth PO1 2UP, UK

**Keywords:** microbiologically influenced corrosion, metal–enzyme hybrids, copper, silver, nanoparticles, sulfate-reducing bacteria, slime-forming bacteria, acid-producing bacteria, antibacterial activity

## Abstract

In search of new materials that would help to prevent microbiologically influenced corrosion (MIC), we have designed and synthetized six different copper and copper–silver nanoparticle–enzyme hybrids using a mild-conditions method carried out in water and r.t. Characterization analyses exhibited the presence of small crystalline nanoparticles with diameters from 2 to 20 nm. X-ray diffraction determined that the Cu hybrids were composed of different copper species, depending on the synthetic protocol used, while the Cu–Ag hybrids were mainly composed of copper and silver phosphate metallic species. Then, the bacterial viability of three MIC-relevant enrichments, sulfate-reducing bacteria (SRB), slime-forming bacteria (SFB), and acid-producing bacteria (APB), was studied in the presence of the bionanohybrids. The results demonstrated a notable effect of all bionanohybrids against SRB, one of the most prominent bacteria associated with MIC. In particular, **Cu-2** and **Cu**–**Ag-2** showed a reduction in bacterial cells of 94% and 98% after 48 h, respectively, at a concentration of 100 ppm. They also exhibited high efficiencies against SFB, with **Cu**–**Ag-1** and **Cu**–**Ag-2** hybrids being the best, with bacterial reduction percentages of 98% after 45 h of exposition at a concentration of 100 ppm. However, in the case of APB, the effect of the hybrids was lost due to the low pH level generated during the experiment. Finally, the capacity of **Cu-2** and **Cu**–**Ag-2** to inhibit the adhesion of SRB to the surface of carbon steel coupons was evaluated. Fluorescence imaging of the surface of the coupons at 24 h demonstrated that the presence of the hybrids inhibited the growth of SRB, obtaining a maximum reduction of 98% with **Cu-2**. Overall, the results of this study demonstrate that these novel nanomaterials have a wide-range antibacterial effect and may have a promising future in the prevention and treatment of MIC.

## 1. Introduction

Microbiologically influenced corrosion (MIC) is a global phenomenon that mainly affects metal surfaces exposed to almost all types of environments. Many definitions have been given for MIC over the years. One of the most recent ones defines MIC as “the corrosion affected by the presence or activity, or both, of microorganisms” [1].

Since the beginning of MIC research, sulfate-reducing bacteria (SRB) have been associated with MIC and identified as an indicator of its presence [2]. In addition, over the years, other bacterial groups have also demonstrated their influence on this type of corrosion, such as slime-forming bacteria (SFB), which generate extracellular polymeric substances (EPS), or acid-producing bacteria (APB) [2,3]. Other types of micro-organisms have also been associated with MIC, such as algae, protozoa, diatoms, archaea, and fungi, but bacteria stand out as the most influential, and particularly SRB [4,5,6]. Different strategies have been developed to avoid MIC, including mechanical methods, such as biofilm surface cleaning, or chemical, like the use of corrosion inhibitors or biocides [1]. In particular, the use of compounds that inhibit the formation of biofilms, which adhere to the metal surface, or the use of antimicrobial agents in the system may be used [3,7].

In search of new materials that can efficiently, safely, economically, and sustainably combat bacteria, such as a biocide or in a form of a coating on a surface, new bionanomaterials with antimicrobial properties have been developed in recent years [8,9,10]. Indeed, carbon-based nanomaterials such as graphene or carbon nanotubes and organic-based nanomaterials, including dendrimers, cyclodextrin, liposomes, and micelles, have been recently identified as potent candidates for microbial growth inhibition [11]. Different metal nanoparticles, including iron and iron oxide nanoparticles [12], Ag, ZnO, or copper nanoparticles [11], have also shown promising results as biocorrosion inhibitors. In terms of the antimicrobial properties, the metal nanoparticle size, morphology, and the aggregation state have been described as critical issues [13].

Therefore, methodologies that can control the size and morphology while preventing the aggregation of nanoparticles seem to be mandatory to enhance their catalytic efficiency. This is particularly important in processes like the Fenton reaction, which generates reactive oxygen species (ROS), one of the ways to kill bacteria. A strategy recently described with successful results in terms of obtaining new potential catalytic nanomaterials is based on the use of an isolated enzyme, such as a biological agent, as scaffold for the in situ formation of metal nanoparticles directly from metal salt [14,15]. In addition, the enzyme has an important role in the final biohybrid formed as a network that stabilizes the nanoparticles, allowing the nanoparticles to be in a well-homogenized distribution around the protein net, avoiding aggregation, a critical point in order to have an accessible reactive nanoparticle surface. Moreover, the enzyme structure allows us to control the final size and shape, producing very small nanoparticles.

Recently, this type of system has also been shown to inhibit several species of viruses and bacteria [16,17,18].

Regarding the antimicrobial mechanisms of these MeNPs–enzyme hybrids, they could act in two different ways: in the extracellular or intracellular environment of the cells. In the extracellular environment, nanoparticles can damage the cell membrane either by directly degrading the compounds that form the membrane or indirectly by generating reactive oxygen species (ROS), which cause membrane damage. In the intracellular environment, MeNPs could inhibit enzymes, induce oxidative stress, and modify the gene expression levels [19,20]. In particular, silver and copper are well known for their antibacterial effects. Some studies suggest that copper primarily acts against bacteria by disrupting the cell membrane and inactivating enzymes [19,21]. In contrast, silver is believed to disrupt cellular functions, damage the cell membrane, target intracellular biomolecules, and induce oxidative stress [22,23,24].

**Figure 1:**
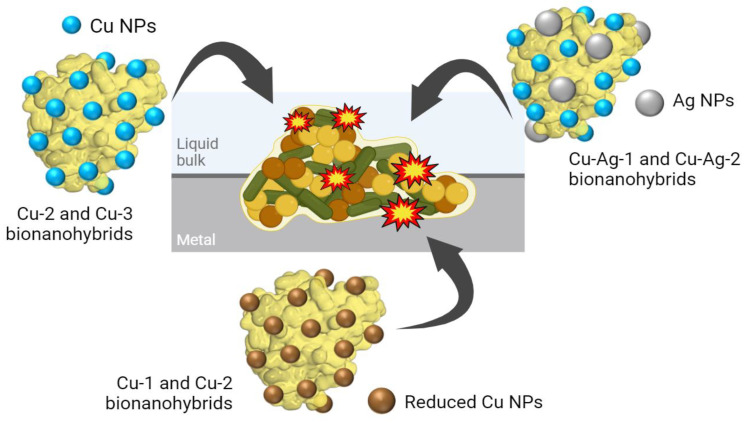
Schematic illustration of bionanohybrids attack against an APB, SRB, and SFB biofilm.

Here, in this work, different monometallic (Cu) and bimetallic (Cu/Ag) nanobiohybrids have been synthesized using the lipase of *Candida antarctica B* (a commercial and very stable enzyme) at room temperature in aqueous media. Then, the antimicrobial efficacy of the different biohybrids was evaluated against different types of MIC bacteria, acid-producing bacteria (APB), sulfate-reducing bacteria (SRB), and slime-forming bacteria (SFB) (Figure 1).

## 2. Materials and Methods

### 2.1. Materials

Copper (II) sulfate pentahydrate, hydrogen peroxide (33% *v*/*v*), sodium hydroxide, sodium dihydrogen phosphate 1-hydrate, and sodium borohydride were obtained from Panreac (Barcelona, Spain). Silver nitrate was obtained from Merck (St. Louis, MO, USA). Lipase B from *Candida antarctica* (CAL-B) solution was supplied by Novozymes (Copenhagen, Denmark).All cultures tested are enrichments from the Endures B.V. strain collection, and their origins are a MIC damage case of an industrial process water pipe of a chemical production site in Belgium. The pipe system had a perforation, and corrosion products were collected from the pipe surface and tested for the presence of MIC-relevant microorganisms. The following three different enrichments were used from this damage case: slime-forming bacteria (SFB), sulfate-reducing bacteria (SRB), and acid-producing bacteria (APB). The bacteria were grown in the corresponding media and the corrosion products from the failure case were used as the inoculum. The enrichments were stored at 4 °C in the Endures in-house strain collection.

### 2.2. Culture Media

SFB culture medium: A total of 5 g of bacteriological peptone from meat and 3 g of meat extract were dissolved in 1 L of demi water and autoclaved.

APB culture medium: A total of 5 g of D-glucose, 10 g of tryptone, 10 g of bacteriological peptone, 1.59 mL of glycerol, 0.2 g of magnesium sulfate heptahydrate (MgSO_4_·7H_2_O), and 0.01 g of phenol red were dissolved in 1 L of demi water and autoclaved. Then, 0.2 g of sodium thiosulfate pentahydrate (Na_2_S_2_O_3_·5H_2_O) and 0.2 g of dipotassium phosphate (K_2_HPO_4_) were dissolved in 50 mL and added to the previous solution by sterile filtration. APB medium changes from red to yellow when the culture is active.

SRB culture medium: First, 1 g of ammonium chloride (NH_4_Cl), 4.5 g of sodium sulfate anhydrous (Na_2_SO_4_), 0.04 g of calcium chloride dihydrate (CaCl_2_·2H_2_O), 0.06 g of magnesium sulfate heptahydrate (MgSO_4_·7H_2_O), 4.2 mL of DL-Na-Lactate, and 1 g of yeast extract were dissolved in 990 mL of demi water and autoclaved.

Second, 4 mg of iron (II) sulfate heptahydrate (FeSO_4_·7H_2_O) was dissolved in 50 µL of H_2_SO_4_. Once completely dissolved, it was added to a third solution, composed of 0.3 g of sodium citrate dihydrate, 0.1 g of L-ascorbic acid, 0.5 g of dipotassium phosphate (K_2_HPO_4_), and 10 mL of demi water. Finally, this mixture was added to the starting solution by sterile filtration. SRB medium changes from yellow to black when the culture is active.

### 2.3. Synthesis of Copper Bionanohybrids

Copper bionanohybrids **Cu-1** and **Cu-2** were prepared by adding 3.6 mL of lipase CAL-B solution (10.34 mg/mL) to 60 mL of sodium phosphate solution (0.1 M, pH 7) in a 250 mL glass bottle containing a small magnetic bar stirrer. After that, 600 mg of Cu_2_SO_4_·5H_2_O (10 mg/mL) was added to the solution and it was stirred for 1 h at room temperature. Then, different quantities of NaBH_4_ were added to the mixture, including 300 mg for **Cu-1** and 30 mg for **Cu-2**. Before the addition, the reducing agent was dissolved in 6 mL of water and it was added to the mixture in 2 times of 3 mL and stirred for 30 min. After the reduction step, the mixture was centrifuged for 5 min at 3000 rpm and the solid obtained in the generated pellet was washed with distilled water. This process was repeated two more times. Finally, the solid was resuspended in 2 mL of distilled water in a cryotube, frozen with liquid nitrogen, and lyophilized for 16h.

The bionanohybrid **Cu-3** was synthetized by adding 1.8 mL of CAL-B to 60 mL of sodium phosphate solution (0.1 M, pH 7) and 600 mg of Cu_2_SO_4_·5H_2_O. The mixture was stirred with a magnetic bar for 1h at room temperature. After that, a light blue emulsion with a concentration of 5000 ppm was obtained, as reported in a previous synthesis protocol for copper bionanohybrids [16]. **Cu-4** was obtained in the same way as **Cu-3** but by finally adding 0.5% (*v*/*v*) of hydrogen peroxide.

ICP-OES analyses were performed by quintupled to determine the metal content in each mg of solid material. The content of Cu (in wt.%) in each case was 45.2 ± 0.22 in **Cu-1**, 27.4 ± 0.3% in **Cu-2**, 33.3 ± 0.4% **Cu-3,** and 32.0 ± 0.9% in **Cu-4**.

### 2.4. Synthesis of Copper–Silver Bionanohybrids

Copper–silver bionanohybrids were generated by adding different amounts of silver nitrate to the **Cu-3** hybrid using different media. First, 20 mL of **Cu-3** was centrifuged and washed three times with distilled water, and the supernatant was removed. **Cu–Ag-1** was prepared by adding 20 mL of sodium phosphate solution (0.1 M, pH 7) to the washed **Cu-3** pellet and 240 mg of silver nitrate salt. **Cu–Ag-2** was synthetized by adding 20 mL of distilled water and 12 mg of silver nitrate to the washed **Cu-3**. After that, in both cases, the mixture was stirred for 24 h at room temperature and, after that, it was centrifuged for 8 min at 3000 rpm. The supernatant was removed, the pellet was washed with distilled water, and this process was repeated three times. Lastly, the solid was resuspended in 2 mL of distilled water in a cryotube, frozen with liquid nitrogen, and lyophilized for 16 h.

ICP-OES analyses were performed by quintupled to determine the metal content in each mg of solid material. The content (in wt.%) was 7.6 ± 0.8% of Cu and 31.7 ± 1.2% of Ag in Cu–Ag-1 and 32.1 ±1.2% of Cu and 3.7 ± 0.6 of Ag in Cu–Ag-2.

### 2.5. Bionanohybrid Characterization Techniques

Inductively coupled plasma-optical emission spectrometry (ICP-OES) was performed using an OPTIMA 2100 DV instrument (PerkinElmer, Waltham, MA, USA). X-ray diffraction (XRD) patterns were obtained using a Texture Analysis D8 ADVANCE Diffractometer (Bruker, Billerica, MA, USA) with Cu K*α* radiation. Their analysis was performed using the X’Pert Highscore Plus programs. Transmission electron microscopy (TEM) images were obtained using a 2100F microscope (JEOL, Tokyo, Japan) equipped with an EDX detector INCA x-sight (Oxford Instruments, Abingdon, UK).

### 2.6. Bacteria Growth

Bacterial cultures of SFB, APB, and SRB, isolated from industrial process water environments, were grown in the appropriate culture media in a final volume of 25 mL from the previous cultures using inoculums of 10%. The incubation was aerobic for SFB and anaerobic for SRB and APB. In the preparation of the SRB and APB cultures, anaerobic bottles closed with a Teflon rubber septum were used. After that, a current of N_2_ was flushed to remove the oxygen, and the bottles were kept at room temperature without agitation. For the aerobic strain, SFB, tubes were used for the growth, which were kept at room temperature in an orbital shaker.The growth curves or SRB, APB, and SFB were studied using different initial concentrations of bacteria in the growths, as follows: 1·10^6^, 1·10^7^, and 1·10^8^ cells/mL (Figures S1–S3). After that, 1·10^7^ cells/mL of the initial concentration was selected as the optimum amount for the following experiments.

### 2.7. Cell Counts

To obtain the bacterial cell/mL concentration, a Thoma Cell Counting Chamber (Paul Marienfeld, Lauda-Königshofen, German), and a Leica DM500 Microscope (Leica, Wetzlar, German) with a 40X objective, along with the equation below, were used, following a standard procedure designed by Endures B.V.

(1) $$Concentration\left(\frac{cells}{\mathrm{mL}}\right)=\frac{total\;nº\;cells\;counted*250,000\;}{\;nº\;of\;squares}*dilution\;factor$$

### 2.8. Antibacterial Activity of Bionanohybrids

The bacterial viability in the presence of the bionanohybrids was assessed by adding 50 or 100 ppm of the bionanohybrids to the bacterial solutions with a concentration of 1·10^7^ cells/mL. The mixture was maintained at room temperature in an orbital shaker. Samples were taken at different times, from 0 to 70 h, with 3 replicas per point, to follow the evolution of the growth curves.

The percentage of bacteria reduction was calculated as follows:

(2) $$\begin{array}{c} \%\\ reduction \end{array}=1-\frac{concentration.after\;4\;h}{control\;concentration\;after\;4\;h}*100$$

### 2.9. Coupons Experiments

Carbon steel coupons (2 × 2 cm^2^) were exposed to SRB cultures for early biofilm experiments. The coupons were placed in the bottom of glass bottles with 20 mL of SRB medium, an adequate bacterial volume to obtain a concentration of an order of magnitude of 7 and 50 ppm of the selected bionanohybrid. The experiment was performed in an anaerobic chamber to avoid the presence of oxygen, and the samples were collected after 0 and 24 h of exposure.

To analyze the bacterial attachment to the surface of the coupons and their viability, the surfaces were stained with a LIVE/DEAD^®^ BacLight™ Bacterial Viability Kit (Thermo Fisher, Waltham, MA, USA) for 20 min in the dark. After that, the coupons were carefully rinsed, dried, and preserved in the dark. For the fluorescence imaging, an Olympus BX51 Fluorescence Microscope (Olympus Corporation, Tokyo, Japan) was used.

Each experiment was performed in triplicated.

## 3. Results and Discussion

### 3.1. Synthesis and Characterization of Bionanohybrids

A variety of synthesis strategies have been employed to generate the designed bionanohybrids, starting from a copper bionanohybrid and subsequently incorporating a reducing agent, an oxidizing agent, or a silver salt, in order to search for new sustainable antimicrobials that are able to combat the MIC.

The Cu hybrids were synthesized by mixing an aqueous solution of lipase B from *C. antarctica* (CAL-B) with a buffer phosphate 0.1 M at pH 7 and a copper sulfate salt. After that, **Cu-1** and **Cu-2** bionanohybrids were produced by reducing the mixture using sodium borohydride as a reducing agent (4 mg/mL and 0.5 mg/mL, respectively). In addition, **Cu-3** was directly obtained from the original mixture without using any reducing agent. Moreover, **Cu-4** was generated by adding 0.5% (*v*/*v*) of H_2_O_2_ to the final **Cu-3**.

Cu–Ag bionanohybrids were formed by the addition of different silver nitrate solutions to **Cu-3**, which was previously washed and liquid-removed. **Cu–Ag-1** was produced by the addition of 12 mg/mL of silver nitrate prepared in buffer phosphate 0.1 M at pH 7, while **Cu–Ag-2** was generated by adding 1 mg/mL of silver nitrate in distilled water to the generated **Cu-3** pellet.

**Figure 2:**
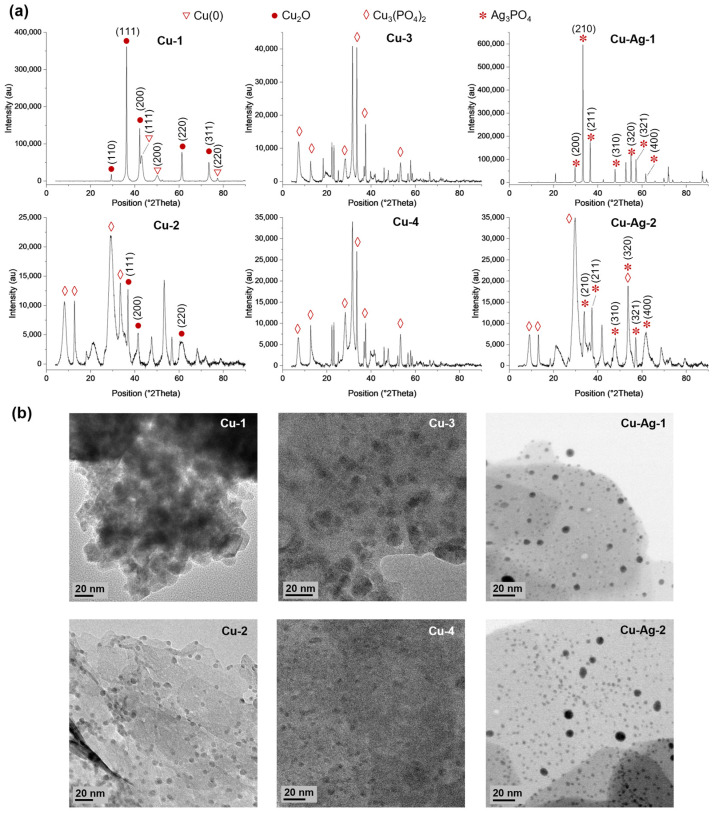
(**a**) XRD analyses and (**b**) TEM image s of bionanohybrids.

Then, XRD analyses were performed to determine the metallic species present in the different hybrids (Figure 2a). The peaks (110), (110), (200), (220), and (311) found in **Cu-1** exhibited a high degree of correlation with the Cu_2_O standard data (JCPDS card 05-0667), while the peaks (111), (200), and (220) matched well with the JCPDS card 04-0836, which corresponds to Cu(0) [25]. **Cu-2** presents peaks that correspond to the Cu_2_O standard, which were located in two theta positions of 36.8, 41.4, and 60.3. However, larger peaks that match well to the Cu_3_(PO_4_)_2_ standard data (JCPDS card no 00-022-0548) were observed. In **Cu-3** and **Cu-4**, the predominant peaks correspond to the Cu_3_(PO_4_)_2_ standard. Furthermore, a higher crystallinity was observed for **Cu-1**, in comparison to **Cu-2**, **Cu-3,** and **Cu-4**, which present a more amorphous structure due to the low peak intensities encountered (Figure 2).

On the other hand, (200), (210), (211), (310), (320), (321), and (400) peaks that match well with the Ag_3_PO_4_ standard (JCPDS card no. 06-0505) were identified in Cu–Ag bionanohybrids, with a higher prevalence of this species being observed in **Cu–Ag-1** than in **Cu–Ag-2**. In addition, peaks corresponding to Cu_3_(PO_4_)_2_ were observed in **Cu–Ag-2** (Figure 2a).

An analysis using Fourier transform infrared (FT-IR) also showed the characteristic peaks of phosphate around 1045 and 985 cm^−1^ in **Cu-2**, **Cu-3**, **Cu-4**, **Cu–Ag-1,** and **Cu–Ag-2**. (Figure S4). The bands at 620 and 556 cm^−1^ correspond to the Cu–O stretching vibration, and they can be observed in **Cu-1** and **Cu-2**, **Cu-3**, **Cu-4**, **Cu–Ag-1,** and **Cu–Ag-2**, being more intense in Cu-1. Thus, it can be seen that these data agree with those obtained using XRD.

Additionally, the average crystallite sizes were determined using the Scherrer method, and they were found to be 18.9, 10.1, 5.0, 7.9, 46.4, and 11.3 nm for **Cu-1**, **Cu-2**, **Cu-3**, **Cu-4**, **Cu–Ag-1,** and **Cu–Ag-2**, respectively.

TEM characterization showed the formation of small, crystalline nanoparticles on the surface of the different bionanohybrids (Figure 2b). The larger NPs were found on **Cu-1**, which showed an average particle diameter size of 11.5 nm, followed by **Cu-2**, with 6.4 nm (Figure S5). **Cu-3** and **Cu-4** showed smaller NPs, with average diameter sizes of 2.9 and 3.0 nm, respectively (Figure S5). In the case of the Cu–Ag hybrids, **Cu**–**Ag-1** showed mainly very large crystalline nanoparticles (Figure S6) with a minor number of smaller NPs of around 15 to 20 nm (Figure 2b), which was confirmed by the high Ag content, as shown by the HAADF-STEM-EDX characterization (Figure S7). **Cu–Ag-2**, with a much lower Ag content, showed the presence of larger NPs corresponding to Ag and also smaller-sized nanoparticles of around 3–3.5 nm, corresponding to the Cu nanoparticles (Figure 2b).

### 3.2. Antibacterial Activity of Bionanohybrids

The antibacterial activity of the bionanohybrids was tested against three different MIC-relevant enrichments isolated from industrial process water environments, all from the Endures B.V. strain collection, as follows: slime-forming bacteria, sulfate-reducing bacteria, and acid-producing bacteria. The free enzyme used in the hybrid fabrication did not show any antimicrobial activity.

#### 3.2.1. Slime-Forming Bacteria

Slime-forming bacteria (SFB) are characterized by the production of a variety of extracellular polymeric substances, or slime, under aerobic conditions. One of their main roles in MIC is to create a favorable environment for anaerobic bacteria, such as SRB, and to protect them from the external environment [3,26].

**Figure 3:**
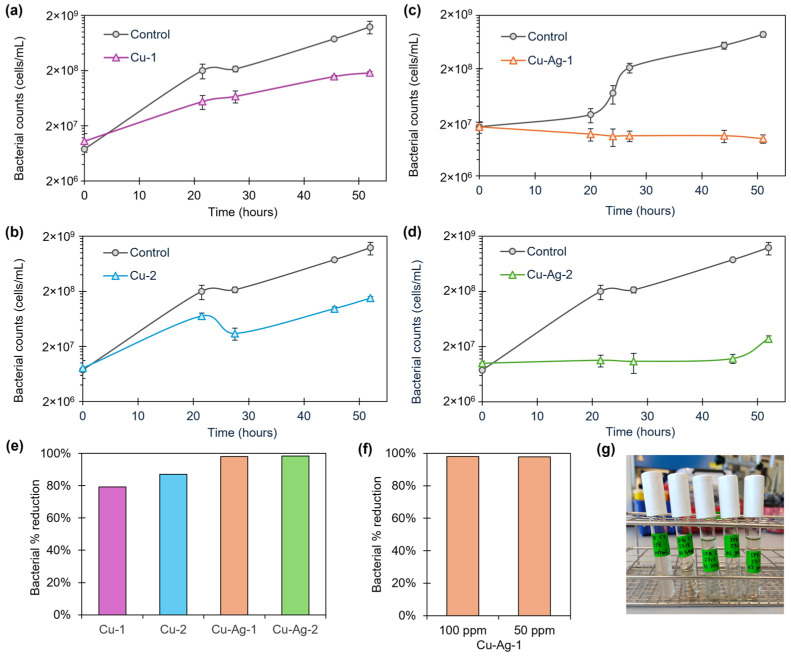
SFB antibacterial assay. Bacterial cell growth from 0 to 52 h with 100 ppm of (**a**) **Cu-1**, (**b**) **Cu-2**, (**c**) **Cu–Ag-1,** and (**d**) **Cu–Ag-2** bionanohybrids. (**e**) Percentage reduction in bacterial cell counts after 45 h of exposure of the different hybrids in a 100-ppm concentration. (**f**) Percentage reduction in bacterial cell counts after 45 h of exposure of **Cu–Ag-1** in 100- and 50-ppm concentration. (**g**) Evolution of the slime content in the test tubes after 2 days of exposition. Bacterial growth curves show the average of three replicas. Error bars correspond to the standard deviation.

The Cu and Cu–Ag bionanohybrids were tested in a 100-ppm concentration against a starting SFB solution of 1·10^7^ cells/mL. The growth of SFB was followed by taking different sampling points between 0 and 52 h. A decrease in the bacterial population was found on the SFB growth, especially after 24 h, compared to the controls (Figure 3). Percentage reductions in the cell count of 80%, 87%, 98%, and 98% were found for **Cu-1**, **Cu-2**, **Cu–Ag-1,** and **Cu–Ag-2**, respectively, after 45 h of exposition (Figure 3e).

After that, **Cu**–**Ag-1**, one of the hybrids with the best antibacterial effect in the previous test, was selected to perform an extra experiment reducing the concentration to 50 ppm (Figure S8). The antimicrobial capacity was found to be maintained, reaching a percentage reduction of 98% after 45 h of contact (Figure 3f). This showed that reducing the concentration of the hybrid to 50 ppm had no effect on the antibacterial performance.

On the other hand, the visual observation of the test tubes where the experiment was conducted indicated a notable reduction in the slime content, being visually undetectable after two days of exposure, compared to the control (Figure 3g).

The results demonstrate that the copper–silver nanohybrids, with a reduction in SFB cells of 98% in both cases after 45 h, are more effective against SFB than the copper nanohybrids. Furthermore, the differences in the SFB inhibition between **Cu–Ag-1** and **Cu–Ag-2** were minimal. This suggests that the incorporation of silver into the copper bionanohybrid enhances the antibacterial effect against SFB. On the other hand, **Cu–Ag-1** had a considerably higher silver content than **Cu–Ag-2** (20% higher). This indicates that the elevated silver quantities in the hybrid composition do not improve its antibacterial efficacy.

#### 3.2.2. Sulfate-Reducing Bacteria

Sulfate-reducing bacteria (SRB) do not require oxygen for growth and activity. They consume sulfates during the respiration process and produce sulfides, which can lead to corrosion and environmental issues [3,11]. As a result, they are of particular concern in the battle against MIC.

**Figure 4:**
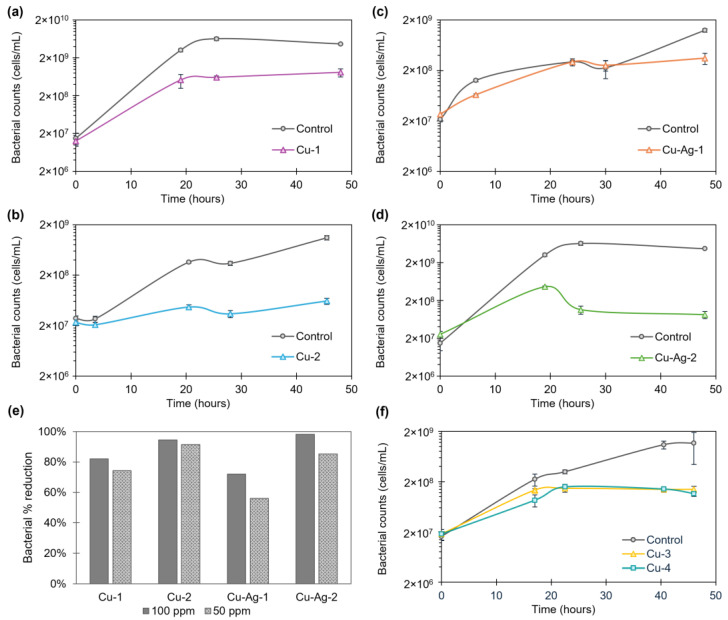
SRB antibacterial assay. Bacterial cell growth from 0 to 48 h of SFB with a concentration of 100 ppm of (**a**) **Cu-1**, (**b**) **Cu-2**, (**c**) **Cu–Ag-1**, and (**d**) **Cu–Ag-2** bionanohybrids. (**e**) Percentage reduction in bacterial cell counts after 48 h of exposure of the different hybrids in a 100-ppm and 50-ppm concentration against SFB. (**f**) Bacterial cell growth from 0 to 46 h of SFB with 50 ppm of **Cu-3** and **Cu-4**. Bacterial growth curves show the average of three replicas. Error bars correspond to standard deviation.

In the first step, **Cu-1**, **Cu-2**, **Cu**–**Ag-1**, and **Cu**–**Ag-2** hybrids were used in a concentration of 100 ppm against SRB (1∙10^7^ cells/mL), following the cell growth from 0 to 48 h (Figure 4a–d). In all cases, an inhibition in SFB growth was demonstrated, but it was more pronounced with the hybrids **Cu-1**, **Cu-2,** and **Cu–Ag-2**, particularly after 24 h. Following a 48-h period, the percentages of bacterial reduction in the cell counts of 82%, 94%, 72%, and 98% for **Cu-1**, **Cu-2**, **Cu–Ag-1,** and **Cu–Ag-2**, respectively, were achieved.

Then, the concentration of the hybrids was reduced to 50 ppm, and the growth curves were obtained (Figure S9). In this instance, the growth of SRB was slightly less inhibited than that observed at 100 ppm. Nevertheless, the values reached with the hybrids were lower than those observed in the controls, with the best inhibition values being exhibited by **Cu-2** and **Cu–Ag-2**. Percentages of bacterial reduction of 74%, 92%, 56%, and 85% were obtained, respectively, for **Cu-1**, **Cu-2**, **Cu–Ag-1**, and **Cu–Ag-2** after 48 h of exposure.

After that, **Cu-3** and **Cu-4** were tested in a concentration of 50 ppm against SRB, due to the good results that the copper hybrids had demonstrated in the previous experiment. Samples were collected at different intervals between 0 and 46 h, and the SRB growth profiles shown in Figure 4f were obtained. They showed growth inhibition compared to the control, with bacterial reduction percentages of 88% and 90% for **Cu-3** and **Cu-4** at 46 h, respectively.

The results indicate that the bionanohybrids tested showed antibacterial activity against SRB in a concentration of 100 and 50 ppm. In addition, decreasing the concentration to 50 ppm generated bacterial reduction percentages of up to 92% after 2 days of exposure for the best of them, **Cu-2**. Among the copper–silver hybrids, **Cu–Ag-2** showed the best results, with bacterial reductions of 98% and 85% after 48 h with a concentration of 100 ppm and 50 ppm, respectively. In this way, modifications in the synthesis processes have led to differences in the structure of the bionanohybrids designed that play a key role in their efficiency.

#### 3.2.3. Acid-Producing Bacteria

Acid-producing bacteria (APB) generate organic acids when growing under certain conditions, including the absence of oxygen. These low pH conditions facilitate the corrosion of metal surfaces. This group of bacteria is normally associated with SRB [2,3].

**Figure 5:**
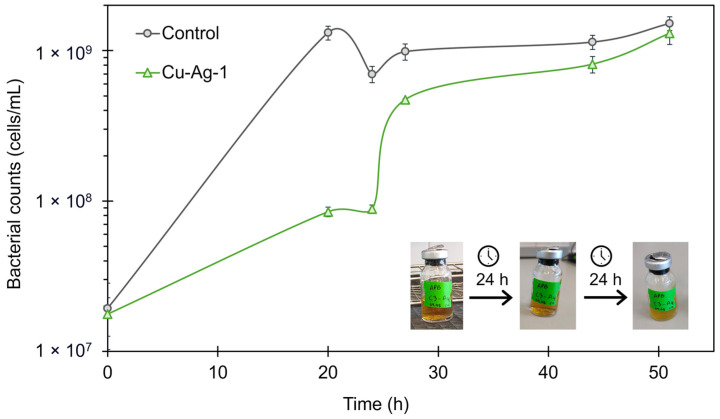
Bacterial cell growth from 0 to 52 h of APB with the **Cu–Ag-1** bionanohybrid in a 100-ppm concentration.

The **Cu–Ag-1** bionanohybrid was selected to be used against APB, in a concentration of 100 ppm. According to the growth curves obtained, as is shown in Figure 5, the growth of APB was inhibited by **Cu–Ag-1** in the first stage of the curve, with a percentage of cell reduction of 94% at 20 h. However, after 24 h of exposition, the concentration of APB cells grew strongly, and, 48 h later, it almost reached the control concentration.

During the experiment, pH measurements were taken at 24 and 48 h. After 24 h, the control showed a pH of 4, while the sample exposed to **Cu–Ag-1** had a pH of 6. The bacterial reduction percentage obtained for the hybrid at that point was 87%. However, after 48 h, the pH values were 4 for both solutions, and the bacterial growth curve for **Cu–Ag-1** indicated that the bacterial community had returned to the control levels.

Moreover, the color of the APB culture medium, which changes from red to yellow when the bacteria are active, also indicated that the bacterial activity persisted after the addition of the bionanohybrid at 48 h, as is observed in Figure 5. Thus, the decrease in the pH value and the color of the culture suggested that the acids and the low pH generated by APB made the bionanohybrids unstable, so their effect disappeared, and the bacteria grew back to the control levels.

Therefore, the remaining hybrids were not used for testing against APB, as they are not suitable for controlling this type of bacteria, and a new design is necessary so that they can withstand the low pH generated by APB.

### 3.3. Coupons Experiments

In the final step of this work, the capacity of the bionanohybrids to inhibit SRB adhesion to a metal surface and prevent the resulting corrosion was evaluated. Carbon steel coupons were used as the metal surface, and **Cu-2** and **Cu–Ag-2** were selected for this experiment at a concentration of 50 ppm, as they had demonstrated the best performance against SRB.

**Figure 6:**
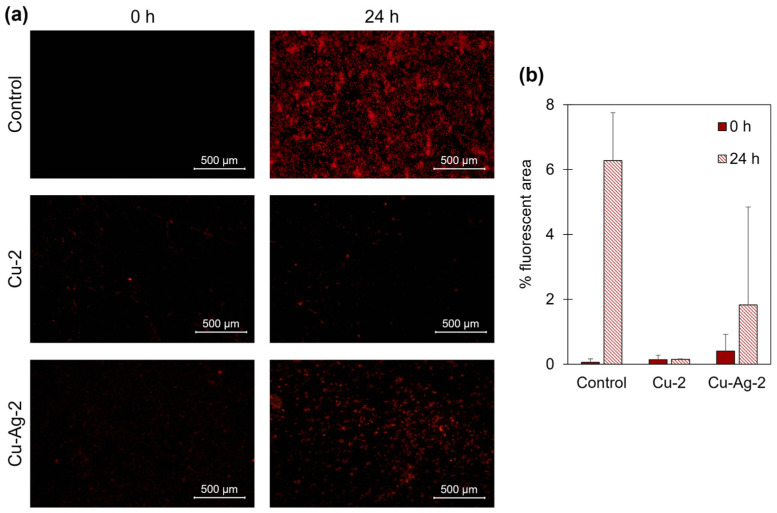
(**a**) Representative fluorescence microscope images of carbon steel coupons treated with **Cu-2** and **Cu–Ag-2** bionanohybrids for 24 h in a 50-ppm concentration. (**b**) Percentage of fluorescence area in the coupons after 0 and 24 h.

The metal coupons were submerged in a solution of SRB with an average concentration of 1∙10^7^ cells/mL and the selected hybrids under anaerobic conditions. Samples were taken after 0 and 24 h. Then, the coupons were removed from the media and stained for fluorescent imaging. The analysis of the fluorescence images after 24 h revealed a minor bacterial growth on the surface of the coupons that were in contact with the bionanohybrids, compared to the controls (Figure 6a). The percentage of fluorescence area of the control samples indicated a bacterial occupation of 6.3% after 24 h. However, the fluorescent area percentage for the **Cu-2** and **Cu–Ag-2** samples was 0.1% and 1.8%, respectively (Figure 6b).

These data indicate that the presence of the bionanohybrids effectively inhibits the growth of SRB on the surface of the coupons after 24 h. This approach has demonstrated the efficacy of **Cu-2** and **Cu–Ag-2** bionanohybrids in the reduction in bacteria concentration on the surface of the metal coupons with the use of small concentrations of the hybrids of only 50 ppm. The most efficient results were observed with **Cu-2**, where it was demonstrated that the percentage of bacterial occupancy on the coupons was reduced by 98%, and the number of bacteria in the aqueous medium was reduced by 98% after 24 h.

Recently, various mechanisms for the antimicrobial action of enzyme–metal biohybrids have been proposed, although they are not yet fully understood. Some studies suggest that these hybrids envelop bacteria, particularly Gram-negative ones, until the interaction becomes so intense that the bacterial membrane collapses, creating the greatest damage in the extracellular environment. In contrast, against Gram-positive bacteria, it seems that the bionanohybrids penetrate the membrane, creating holes and generating the greatest damage to the intracellular environment [27,28]. These studies also highlight the synergistic effect of combining metal nanoparticles with enzymes, showing significantly enhanced antimicrobial activity compared to free enzymes or individual nanoparticles.

On the other hand, the power of reactive oxygen species (ROS) generation, a key factor in antibacterial efficacy, must also be considered. This capability is mainly determined by the metal species of the nanoparticles (NPs) and their size, which can vary greatly. For instance, the ROS generation and, therefore, the antibacterial capacity of Cu(II), which is present in all of the biohybrids, has been widely studied [29,30,31].

In **Cu-2**, the presence of both Cu(II) and Cu(I) species, combined with the small size of the NPs, results in enhanced antibacterial capacity compared to other copper hybrids. However, between **Cu–Ag-1** and **Cu–Ag-2**, despite having the same metal species and similar NP sizes, the presence of large structures in **Cu-Ag-1**, as shown in Figure S6, reduces its antimicrobial power compared to **Cu–Ag-2**.

Therefore, antibacterial capacity is influenced by a combination of factors, including particle size, the type of metal species, and their distribution within the nanocomposite. Optimal conditions include lower aggregation, smaller particle sizes, and metal species that favor ROS generation.

## 4. Conclusions

Different Cu and Cu–Ag bionanohybrids were synthesized by a green methodology and evaluated as antimicrobial agents against MIC bacteria.

In the Cu monometallic hybrids, the presence of Cu(I) as metal species in the hybrid (**Cu-2**) showed the best results against SRB and SFB. However, in the bimetallic hybrids, **Cu–Ag-1** and **Cu–Ag-2** showed similar efficiencies against SFB, but not against SRB, where **Cu–Ag-2** showed a greater efficacy compared to **Cu–Ag-2**. This suggests that increasing the silver content of the bimetallic hybrid does not necessarily increase the antibacterial activity against SRB and SFB; however, in both cases, Ag(I) is the species, but the AgNPs are smaller in **Cu–Ag-2**. The inclusion of silver in the synthesis protocol produced hybrids with a superior performance against SFB compared to copper-only hybrids. However, **Cu-2** exhibited a higher efficacy against SRB compared to **Cu–Ag-2**, as demonstrated in the coupons experiment, suggesting that its composition is more adept at combating this particular strain of bacteria. Furthermore, the efficacy of **Cu-2** was maintained when the amount of hybrid was reduced from 100 to 50 ppm.

The inhibition of SRB adhesion to steel coupons was also successfully tested using **Cu-2**, with a maximum reduction in bacterial adhesion of 98% after 24 h exposure.

## Data Availability

The authors confirm that the data supporting the findings of this study are available within the article.

## Supplementary Materials

The following supporting information can be downloaded at: https://www.mdpi.com/article/10.3390/nano14171376/s1, Figure S1. Growth curves of slime-forming bacteria starting from different concentrations; Figure S2. Growth curves of acid-producing bacteria starting from different concentrations; Figure S3. Growth curves of sulfate-reducing bacteria starting from different concentrations; Figure S4. FT-IR spectrum of Cu and Cu–Ag bionanohybrids; Figure S5. Nanoparticle size distribution of Cu and Cu–Ag bionanohybrids; Figure S6. TEM images of the Cu–Ag-1 hybrid; Figure S7. HAADF-STEM-EDX characterization of the Cu–Ag-1 hybrid; Figure S8. SFB cell growth with **Cu–Ag-2**; Figure S9. SRB cell growth with Cu-1, Cu-2, Cu-3, Cu-4, Cu–Ag-1, and Cu–Ag-2 bionanohybrids.
