# Supplementary material for: Metal Bionanohybrids against Microbiologically Influenced Corrosion (MIC) Consortia
Source: Nanomaterials (Basel). 2024 Aug 23;14(17):1376. doi: 10.3390/nano14171376 (PMC11397077; doi:10.3390/nano14171376)
Supplement: Supplementary file 1 [file nanomaterials-14-01376-s001.zip › nanomaterials-3136418-supplementary.pdf]

nanomaterials

Supporting Information

Metal Bionanohybrids against Microbiologically Influenced Corrosion (MIC) Consortia

Clara Ortega-Nieto [1,2], Maria Salta [2,3,*], Nanni Noël-Hermes [2,*] and Jose M. Palomo [1,*]

1 Instituto de Catálisis y Petroleoquímica (ICP), CSIC, c/Marie Curie 2, 28049 Madrid, Spain; clara.ortega@csic.es
2 Endures B.V., 1781 AT Den Helder, The Netherlands
3 School of Biological Sciences, Faculty of Science and Health, University of Portsmouth, Portsmouth PO1 2UP, UK
* Correspondence: maria.salta@endures.nl (M.S.); nanni.noel@endures.nl (N.N.-H.); josempalomo@icp.csic.es (J.M.P.)

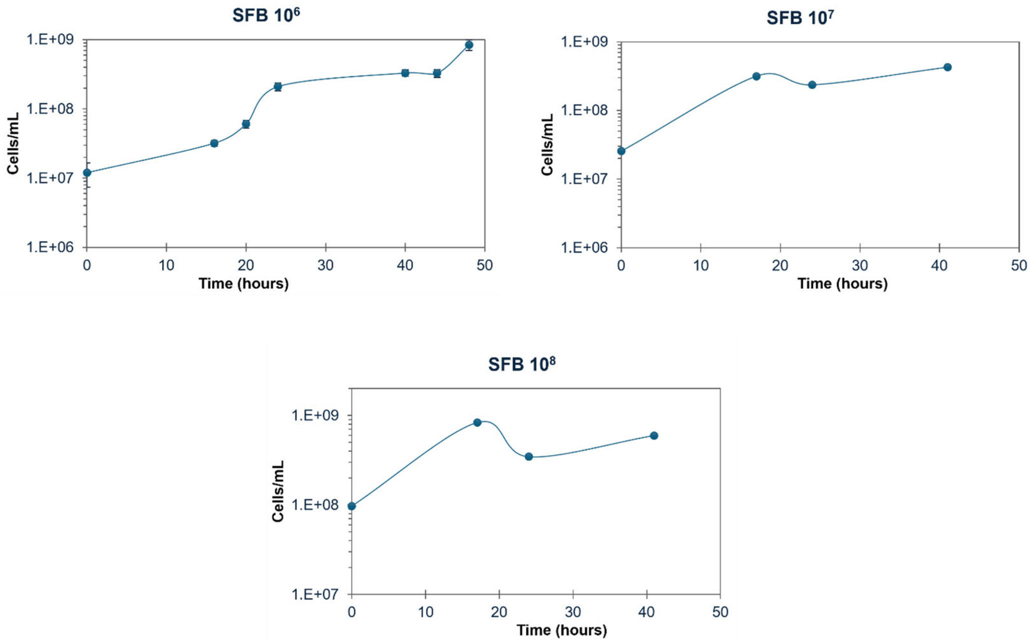

Figure S1. Growth curves of Slime-Forming bacteria starting from different concentrations.

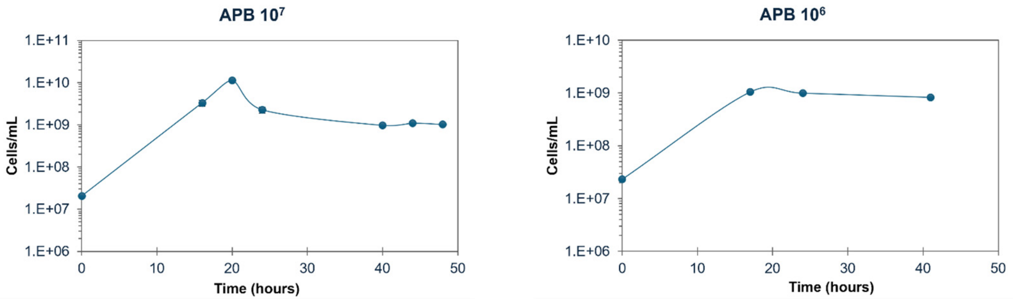

Figure S2. Growth curves of Acid-Producing bacteria starting from different concentrations.

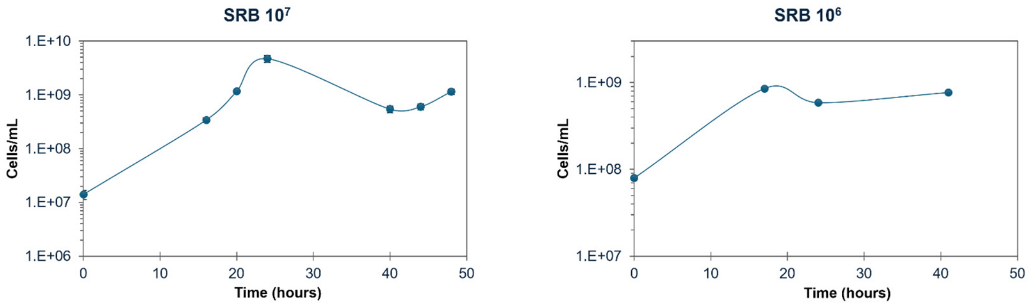

Figure S3. Growth curves of Sulphate-Reducing bacteria starting from different concentrations.

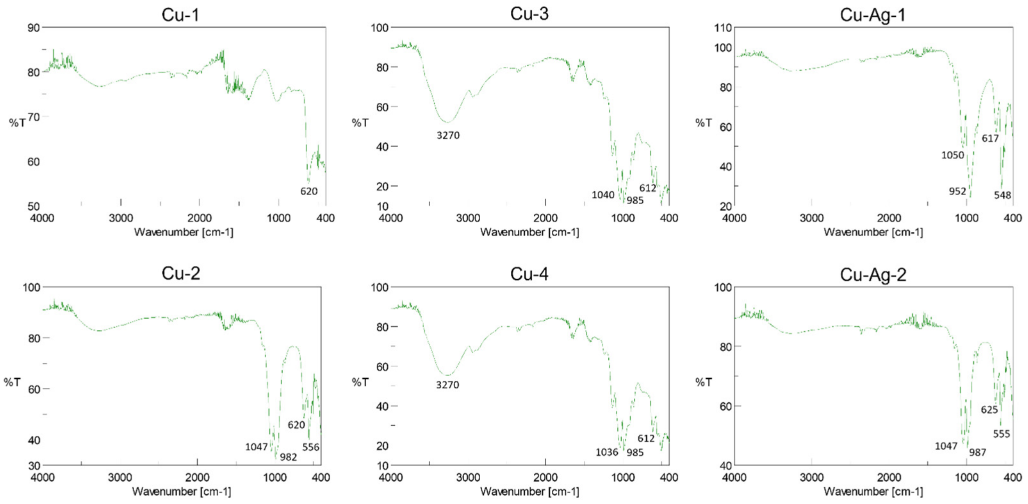

Figure S4. FT-IR spectrum of Cu and Cu-Ag bionanohybrids.

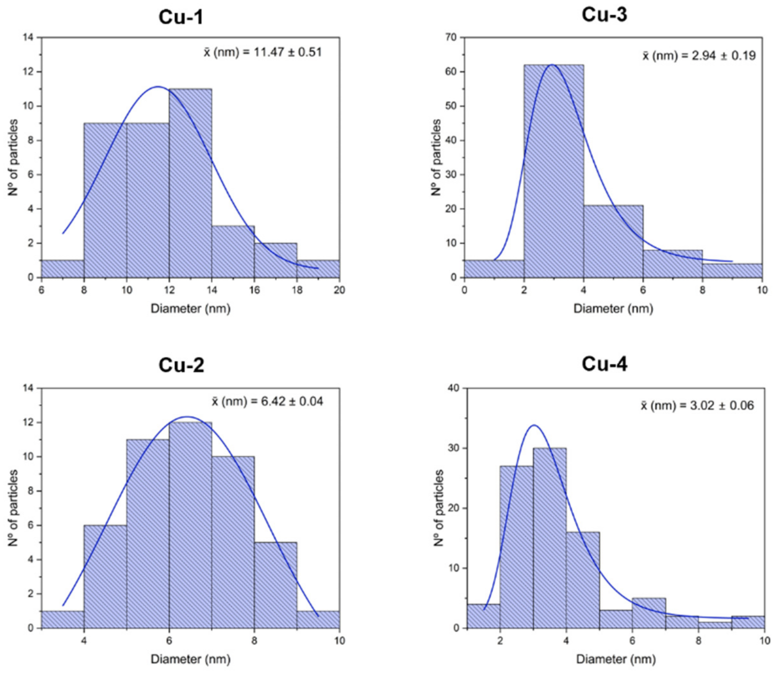

Figure S5. Nanoparticles size distribution of Cu bionanohybrids.

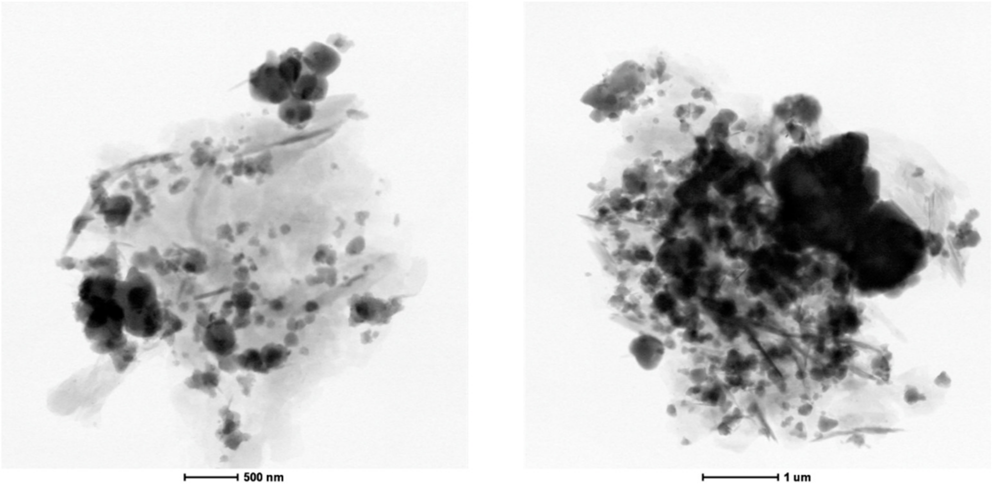

Figure S6. TEM images of Cu-Ag-1 hybrid.

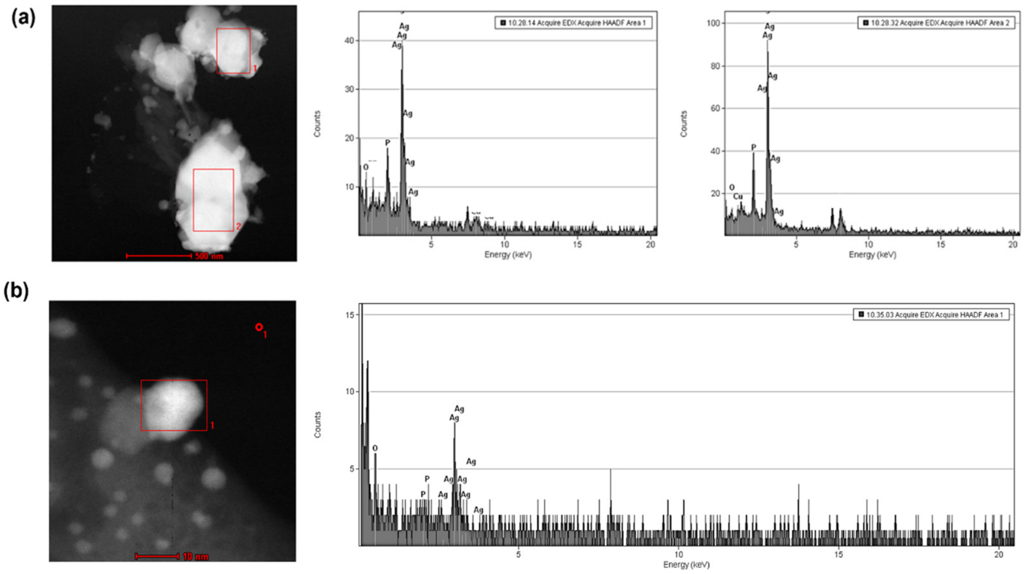

Figure S7. HAADF-STEM-EDX characterization of Cu-Ag-1 hybrid. **(A)** Larger nanoparticles. **(B)** smaller nanoparticles (10–15 nm). The nickel signal corresponds to the grid used.

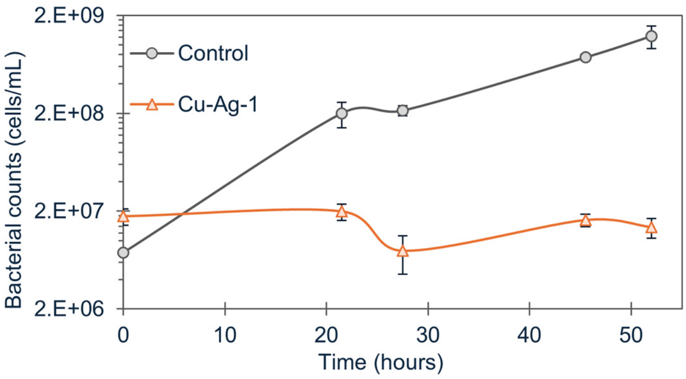

Figure S8. SFB cell growth from 0 to 52 h with Cu-Ag-1 in a concentration of 50 ppm.

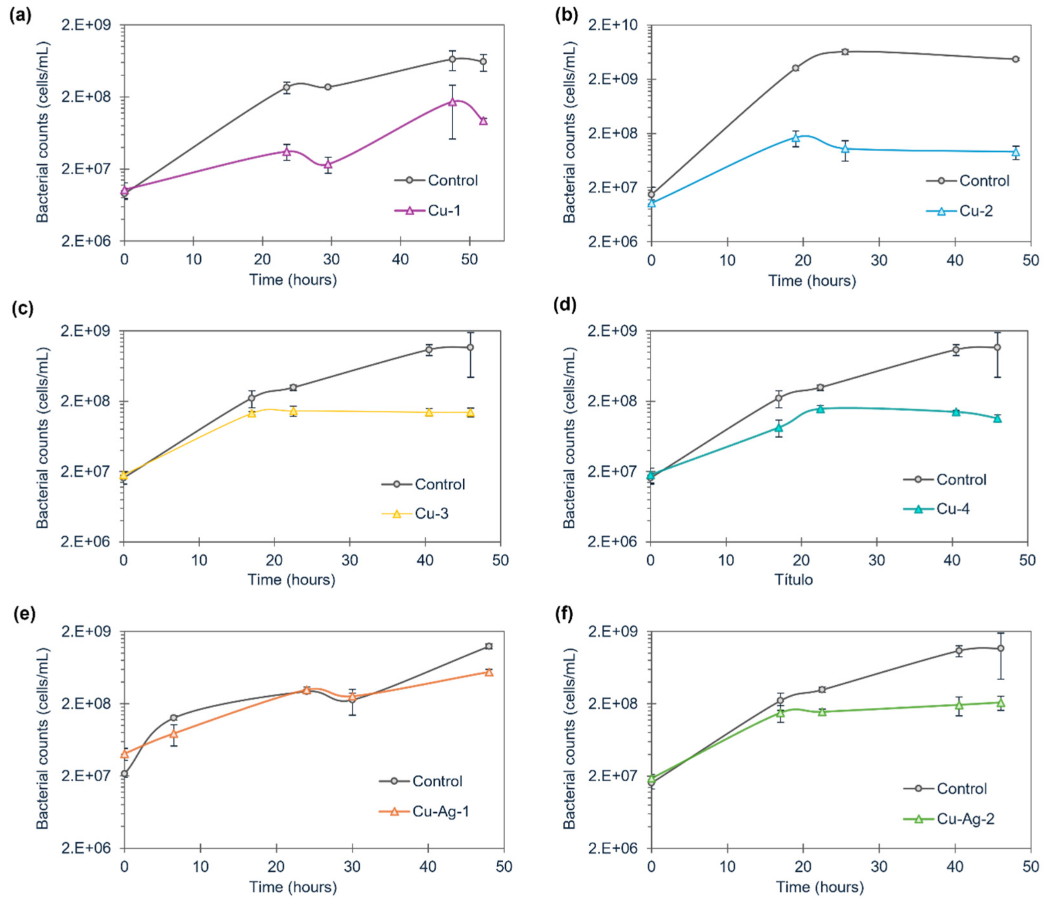

Figure S9. SRB cell growth from 0 to 48 h with a concentration of 100 ppm of **(a)** Cu-1, **(b)** Cu-2, **(c)** Cu-3, (d) Cu-4 **(e)** Cu-Ag-1 and **(f)** Cu-Ag-2 bionanohybrids.
